# Empty Sella Syndrome: long-term impact on pituitary function

**DOI:** 10.25122/jml-2025-0044

**Published:** 2025-08

**Authors:** Latifa Yagoubi, Amal Ourdi, Nisrine Bouichrat, Siham Rouf, Hanane Latrech

**Affiliations:** 1 Department of Endocrinology-Diabetology and Nutrition, Mohammed VI University Hospital Center, Faculty of Medicine and Pharmacy, University of Mohammed 1st, Oujda, Morocco; 2 Laboratory of Epidemiology, Clinical Research and Public Health, Mohammed VI University Hospital Center, Faculty of Medicine and Pharmacy, University of Mohammed 1st, Oujda, Morocco

**Keywords:** Empty Sella Turcica, pituitary insufficiency, gender differences, hypogonadotropic hypogonadism, ESS, Empty Sella Syndrome, CSF, Cerebrospinal Fluid, CT, Computed Tomography, MRI, Magnetic Resonance Imaging, TSH, Thyrotropin, FT4, Free Thyroxine, PRL, Prolactin, FSH, Follicle-Stimulating Hormone, LH, Luteinizing Hormone, IGF-1, Insulin-like Growth Factor 1, GH, Growth Hormone, GHD, Growth Hormone Deficiency, BMI, Body Mass Index, ST, Synacthen Test, ITT, Insulin Tolerance Test

## Abstract

Empty Sella Syndrome (ESS) is characterized by a herniation of cerebrospinal fluid into the pituitary fossa, frequently causing pituitary dysfunction. Although ESS is generally asymptomatic, it can lead to progressive hormonal deficiencies. Long-term studies of pituitary function in patients with ESS are lacking. This study aimed to evaluate pituitary function in ESS patients, monitor the progression of hormone deficiencies, and explore the impact of gender, age, and body mass index (BMI). This is a descriptive and analytical study involving 41 patients diagnosed with ESS and treated at our university hospital between 2014 and 2024. All patients underwent MRI and endocrine testing for cortisol, thyrotropin, free thyroxine, prolactin, growth hormone, gonadal hormones, and testosterone. The median duration of follow-up was 5 years, with a range of 6 months to 10 years. Data was collected and analyzed using SPSS version 21. At diagnosis, 82.9% of patients had at least one pituitary hormone deficiency, primarily in the adrenal, gonadal, and growth hormone axes. Women were more likely to develop gonadal dysfunction (34.1%) than men (21.9%). A positive correlation between age and adrenal/gonadal insufficiency was observed. Additionally, a significant association was found between higher BMI and gonadal insufficiency, highlighting the role of obesity in exacerbating pituitary dysfunction. ESS is commonly linked to pituitary dysfunction, particularly in the adrenal and gonadal axes. Gender, age, and BMI influence the development and progression of hormonal deficiencies, underscoring the need for regular endocrine evaluation and long-term follow-up in these patients.

## INTRODUCTION

Empty Sella Syndrome (ESS) is the most common cause of incidental enlargement of the pituitary cavity, typically detected on radiological imaging [1]. It is characterized by herniation of the subarachnoid space into the sella turcica, resulting in flattening of the pituitary gland against the posteroinferior wall [2]. Although ESS is often benign, it should be distinguished from sellar enlargement caused by pituitary tumors. Some patients with ESS may develop hypopituitarism over time; however, the lack of longitudinal studies limits understanding of its long-term impact and management [3].

ESS is classified as either primary (without prior pituitary pathology) or secondary (following pituitary injury due to surgery, radiation, or other causes) [3]. Primary ESS is thought to result from congenital weakness of the sellar diaphragm, elevated intracranial cerebrospinal fluid pressure, or changes in pituitary gland volume [1]. Secondary ESS is more frequently observed in women and has been associated with obesity, pregnancy, hypertension, headaches, and visual disturbances [4].

This study aimed to evaluate pituitary function in patients with ESS, to monitor the progression of pituitary insufficiency, to assess the potential development of endocrine disorders, and to explore predictive factors that may improve long-term management.

## MATERIAL AND METHODS

### Study design and population

This descriptive and analytical study included 41 patients diagnosed with ESS and treated between August 2014 and August 2024 at the Department of Endocrinology, Diabetology, and Nutrition, Mohammed VI University Hospital, Oujda, Morocco. Patients ranged in age from 7 to 79 years. Both primary and secondary ESS cases were included. Patients with other causes of sellar enlargement, such as pituitary tumors or cysts, were excluded. Informed consent was obtained from all patients or their legal guardians. The study was approved by the local biomedical research ethics committee.

### Study protocol

All patients underwent physical examination, brain magnetic resonance imaging (MRI), as well as neurological and ophthalmological assessments. Collected data included age at diagnosis, sex, body mass index (BMI), history of traumatic brain injury, number of pregnancies (in women), and duration of follow-up. Baseline hormonal evaluation included cortisol, thyrotropin (TSH), free thyroxine (FT4), prolactin (PRL), insulin-like growth factor 1 (IGF-1), growth hormone (GH), luteinizing hormone (LH), follicle-stimulating hormone (FSH), estradiol, and testosterone.

Endocrine deficiencies were defined as follows:

- Central hypothyroidism was identified as low FT4 with low/normal TSH.
- Adrenal function was evaluated using a Synacthen 250 µg test or an insulin tolerance test (ITT), with a cortisol peak ≥18 μg/dL as normal [5].
- Growth hormone deficiency (GHD) was diagnosed by stimulation testing (glucagon-propranolol or ITT), with peak GH <10 mcg/L in children [6].
- Central hypogonadism: in men, defined as low testosterone with low/normal gonadotropins; in women of reproductive age, defined as amenorrhea/oligomenorrhea with low estradiol and low/normal gonadotropins; in postmenopausal women, indicated by non-elevated FSH levels [7].

### Follow-up

Patients were followed every six to 12 months for a median of 5 years (range, 6 months–10 years). Hormone levels were reassessed at each visit, and hormone replacement therapy was initiated as clinically indicated.

### Outcomes

The primary objective was to describe the clinical features of ESS at diagnosis and to assess the need for endocrine testing in incidentally discovered cases. The secondary objective was to evaluate the progression of pituitary hormone deficiencies over time and to determine the incidence of newly developed deficiencies after at least 6 months of follow-up.

### Statistical analysis

Data were analyzed using IBM SPSS Statistics, version 21 (IBM Corp., Armonk, NY, USA). Student’s *t-test* was applied to continuous variables (e.g., age), and the chi-square test was used for categorical variables (e.g., sex, presence of hormone deficiencies). Multivariate logistic regression was performed to identify risk factors (age, BMI, sex) associated with pituitary dysfunction. A *P* value <0.05 was considered statistically significant.

## RESULTS

In this study, 41 patients with ESS were evaluated. The mean age at diagnosis was 39.5 ± 19.9 years. The cohort included 30 women and 11 men. Men were diagnosed at a younger age (mean, 40.0 years) compared with women (mean, 46.1 years), although this difference was not statistically significant (*P* = 0.19). The highest incidence was observed in the 30-50-year age group. Primary ESS was diagnosed in 21 patients (51.2%) and secondary ESS in 20 patients (48.8%). Among the secondary ESS cases, six patients (15%) had a history of Sheehan syndrome, 13 patients (31.7%) underwent previous surgical intervention of the pituitary gland, and one patient (2.4%) had a post-radiation therapy history.

MRI confirmed ESS in all 41 patients, demonstrating features such as enlargement of the sella turcica and flattening of the pituitary gland. In primary ESS, these findings were generally less pronounced; 78% of cases showed a partially empty sella with a thin but visible pituitary gland and no other sellar abnormalities.

ESS was diagnosed incidentally in 22 patients (53.7%) and based on clinical suspicion in 19 (46.3%). Incidental diagnoses were more frequent in men. Overall, 24.4% of patients were obese. Among women, 20% had a history of miscarriage and 15% experienced preterm birth. Hypertension and diabetes mellitus were present in 14.6% and 12.2% of the cohort, respectively.

At diagnosis, 82.9% of ESS patients (*n* = 34) had at least one pituitary hormone deficiency. The majority (63.4%) had combined hormone deficiencies, including cortisol, growth hormone, gonadal hormones, and thyroid hormones. Isolated deficiencies were less common (7.3% cortisol, 2.4% thyroid). Multiple deficiencies were more frequent in secondary ESS (85%), particularly growth hormone deficiency, which was significantly more common than in primary ESS (*P* = 0.02). All Sheehan syndrome patients had both gonadal and adrenal insufficiency.

During a median follow-up of 5 years (range, 6 months–10 years), 38.2% of patients developed progressive pituitary dysfunction, predominantly affecting the adrenal and gonadal axes. Pituitary function remained stable in 51.2%, while 17% showed improvement with hormone replacement therapy. Progression was more frequent in secondary ESS, with 45% of Sheehan syndrome patients demonstrating deterioration.

Across the cohort, multiple pituitary axes were affected. In the adrenal axis, 34% of patients had normal cortisol levels at diagnosis; however, 4.8% developed secondary adrenal insufficiency over 5 years, reflecting gradual adrenal decline. Thyroid function remained stable in most patients, though 4.8% developed hypothyroidism after 3–4 years of follow-up. Hypogonadotropic hypogonadism was documented in 34.1% of women and 21.9% of men. Growth hormone deficiency was identified in 21.9%, particularly among older patients.

Subgroup analysis revealed that gonadal dysfunction was significantly more frequent in women (*P* = 0.03). Increasing age was significantly associated with adrenal (*P* = 0.002) and gonadal insufficiencies (*P* = 0.02), but not with thyroid dysfunction. Elevated BMI was significantly associated with gonadal insufficiency (*P* = 0.03), though no association was found with adrenal or thyroid function.

## DISCUSSION

ESS has a reported prevalence ranging from 8% to 35%, with the widespread use of MRI increasing the detection of asymptomatic cases [8,9]. Because it is frequently discovered incidentally, its diagnosis raises important questions regarding the need for baseline endocrine evaluation and long-term follow-up. In our cohort, the mean age at diagnosis was 39.5 years, with a female predominance, supporting the role of gender-related factors in ESS pathogenesis [10,11]. A study by Atci *et al*. reported a similar median age of onset for ESS [12]. These findings may suggest that hormonal changes throughout reproductive years may play a role in ESS pathogenesis [9,12]. About half (46.3%) of patients were diagnosed based on clinical and biochemical suspicion of pituitary disease, more commonly in men, while the rest (53.7%) were diagnosed incidentally. This supports findings by Akkus *et al*. [13], suggesting fewer endocrine symptoms in men, which may contribute to underdiagnosis and reflect gender differences in ESS presentation [14].

The high prevalence of pituitary insufficiency was one of the main findings of our study, as 82.9% of patients had at least one hormone deficiency at diagnosis. The adrenal and gonadal axes were most often involved, with 63.4% showing combined deficiencies. In agreement with Atci *et al*. [12], our findings show that secondary ESS is associated with a higher prevalence of hormone deficiencies, and only growth hormone deficiency showed a statistically significant difference between primary and secondary ESS (*P* = 0.02).

During a median follow-up of 5 years, 38.2% of patients developed progressive declines in pituitary function, particularly involving the adrenal and gonadal axes. In contrast, 51.2% maintained stable pituitary function, underscoring the heterogeneous course of ESS, as also noted by Sage *et al*. [15]. Notably, 17% of patients demonstrated recovery of pituitary function after hormone replacement therapy, supporting its therapeutic role, in agreement with Atci *et al*. [12].

Sex-specific differences were also evident: women were more likely to experience gonadal dysfunction (34.1% vs. 21.9% in men), potentially related to hormonal fluctuations in estrogen and progesterone. Adrenal and thyroid insufficiencies, however, showed no significant sex differences. Increasing age correlated positively with both adrenal (*P* = 0.002) and gonadal insufficiencies (*P* = 0.02), emphasizing the importance of age-specific monitoring [13]. Higher BMI was also associated with gonadal dysfunction, supporting the role of obesity in hypothalamic-pituitary-gonadal axis dysregulation, though no significant correlation was found with other axes [16]. Hypertension and diabetes were found in 14.6% and 12.2% of the participants, respectively. Although no direct correlation was found between these comorbidities and pituitary dysfunction, their possible role as confounding factors needs further exploration.

Our study has several limitations, including its moderate sample size, retrospective design, and single-center setting, which may limit generalizability. Larger, prospective, multicenter studies with control groups are needed to validate these findings and further elucidate the long-term course of ESS.

## CONCLUSION

This study provides valuable insights into the clinical presentation and endocrine dysfunction associated with ESS. Our findings highlight the high prevalence of pituitary insufficiency, sex-related differences in hormonal deficits, and the influence of age and obesity on pituitary function. Despite limitations, including the small sample size and retrospective, single-center design, these results underscore the importance of routine endocrine evaluation and long-term monitoring of patients with ESS. Larger prospective multicenter studies are needed to further clarify the natural history of ESS and to optimize management strategies.

## Data Availability

The datasets generated and analyzed during the current study are available from the corresponding author upon reasonable request.
